# Music Training Increases Phonological Awareness and Reading Skills in Developmental Dyslexia: A Randomized Control Trial

**DOI:** 10.1371/journal.pone.0138715

**Published:** 2015-09-25

**Authors:** Elena Flaugnacco, Luisa Lopez, Chiara Terribili, Marcella Montico, Stefania Zoia, Daniele Schön

**Affiliations:** 1 Child Neurology and Psychiatry Ward, Institute for Maternal and Child Health - IRCCS ‘‘Burlo Garofolo”, Trieste, Italy; 2 Center for the Child Health - Onlus, Trieste, Italy; 3 Rehabilitation Center for Developmental Disorders, Villaggio Eugenio Litta, Grottaferrata, Rome, Italy; 4 Epidemiology and Biostatistics Unit, Institute for Maternal and Child Health - IRCCS ‘‘Burlo Garofolo”, Trieste, Italy; 5 Aix-Marseille Université, INS, Marseille, France; 6 INSERM, U1106, Marseille, France; MRC Institute of Hearing Research, UNITED KINGDOM

## Abstract

There is some evidence for a role of music training in boosting phonological awareness, word segmentation, working memory, as well as reading abilities in children with typical development. Poor performance in tasks requiring temporal processing, rhythm perception and sensorimotor synchronization seems to be a crucial factor underlying dyslexia in children. Interestingly, children with dyslexia show deficits in temporal processing, both in language and in music. Within this framework, we test the hypothesis that music training, by improving temporal processing and rhythm abilities, improves phonological awareness and reading skills in children with dyslexia. The study is a prospective, multicenter, open randomized controlled trial, consisting of test, rehabilitation and re-test (ID NCT02316873). After rehabilitation, the music group (N = 24) performed better than the control group (N = 22) in tasks assessing rhythmic abilities, phonological awareness and reading skills. This is the first randomized control trial testing the effect of music training in enhancing phonological and reading abilities in children with dyslexia. The findings show that music training can modify reading and phonological abilities even when these skills are severely impaired. Through the enhancement of temporal processing and rhythmic skills, music might become an important tool in both remediation and early intervention programs.

**Trial Registration:**

ClinicalTrials.gov NCT02316873

## Introduction

Developmental dyslexia is a learning disability that affects spelling and print decoding abilities despite normal comprehension, intelligence and adequate education, and in the absence of overt sensory or neurological damage [1]. Dyslexia can be a severely invalidating disorder per se and is a risk factor for increased internalizing problems, anxiety and school failure which can lead to behavioral disorders, especially during adolescence. Its prevalence can be high, ranging from 3% to 10% [1]. Therefore, intervention is crucial not only to improve academic abilities, but also to regulate social interactions.

Across languages, children with dyslexia have poor phonological processing skills, leading to a dominant phonological core deficit [2,3]. Although there are several theories on the causes of this disorder [4], many authors agree that the phonological deficit in dyslexia could be related to a deficit in temporal processing [5]. More precisely, temporal processing may be impaired at several interconnected temporal scales. For instance Hornickel and Kraus [6] found that poor readers have more variable neural responses to speech, possibly leading to an impoverished representation of the fine-structure of speech sound (above 600 Hz) containing the formant patterns that are necessary acoustic cues to place of articulation. On a longer time-scale, of the order of tens of milliseconds, Tallal [7] proposed that the phonological deficit in developmental dyslexia could be due to impaired processing of formant transitions characterizing the phonetic distinctive features of some consonants. At an event longer time scale Goswami and collaborators claimed that amplitude modulations in the envelope, ranging between 2 and 50 Hz, may be one of the critical acoustic properties underlying syllable rate and speech rhythm [8,9]. Interestingly, Amitay and collaborators [10], using an amplitude modulation detection task, found a deficit at slow (4Hz), intermediate (10Hz) and fast modulations (500Hz), showing that there may not be a deficit at a specific temporal scale. The general hypothesis we address in this work is that music training should boost temporal processing at different time scales and this may have in turn a positive impact on phonological awareness and reading abilities in children with dyslexia.

Indeed, music training encompasses a wide range of brain functions, going from sound encoding to higher cognitive functions. There is clear evidence that music training induces functional and structural changes in the auditory and sensori-motor systems, which in turn increases accuracy in music-related tasks [11]. Moreover, the fact that music shares several cognitive functions with other human abilities, for instance language, raises the intriguing possibility that musical expertise transfers to other, not strictly musical domains [12, 13]. Recent data support the view that music training affects speech and language processing. For instance, the group of Nina Kraus has elegantly shown that musicians show a more faithful brainstem representation of linguistic sounds compared to non-musicians. This is reflected, for instance, in a more precise frequency-following response (FFR), finer representation of consonant features [14] and greater resistance to response disruption by noise in both adults [15] and children [16]. Functional differences between musicians and non-musicians have also been observed in the auditory cortex, notably with an enhanced activation pattern for musicians when processing pitch contour in music and speech [17], as well as when processing vowels and consonants [18,19]. Musicians also perform better when distinguishing consonants based on durational cues such as voice onset time [20], formant transition durations and phrasal lengthening [21,22]. They are also more proficient in speech segmentation [23]. Some of these findings have also been extended to children. For instance, musically trained children i) are better at detecting pitch and envelope changes in speech, ii) show higher vocabulary and reading abilities, iii) are more efficient in segmenting a new language (i.e. word extraction from a continuous flow of sounds) and iv) show an overall enhanced verbal intelligence compared to children who do not receive music training [24–27]. One recent possible explanation of this cross-domain transfer, is the precise auditory timing hypothesis (PATH) [28]. According to this model, the enhanced phonological abilities in musicians would be related to the high degree of precision in audio-motor timing required by music, possibly leading to an enhanced perception of the timing of speech sounds.

Notably, several of the temporal processing skills that are enhanced by music training are impaired in reading disorders, leading to the hypothesis that reading disability might be linked to a poor temporal resolution of perceptual systems [8,6,29]. While the evidence of a causal role of music training on reading abilities is scarce [25], several studies have shown that musical perceptual abilities correlate with phonological awareness and reading abilities, and can also be predictive of preschoolers’ reading developmental trajectories [30–33]. While several researchers have hypothesized that there might be a benefit of music training on reading skills in children with dyslexia, so far supporting evidence is scarce [34–37]. Most importantly, this hypothesis has never been addressed using a randomized controlled trial in children with dyslexia—rather than children without other confounding comorbidities-and with a sufficiently long training duration [38].

We report the results of a randomized controlled trial testing the hypothesis that music training, by improving temporal processing at multiple temporal scales, improves phonological awareness and reading skills in dyslexic children.

## Materials and Methods

### Participants Recruitment

All children between 8 and 11 years of age, with a diagnosis of dyslexia, living in the provinces of Trieste and Rome (Italy), who had been referred to the Institute for Maternal and Child Health “Burlo Garofolo”, to Local Health Service (AAS N 1) in Trieste and to the Rehabilitation Center “Villaggio Eugenio Litta” in Grottaferrata, (Rome) during the period from January 1^st^2007 to August 30^th^2011 were checked for eligibility. The study was approved by the Independent Bioethics Committee of the Institute of Maternal and Child Health—IRCCS ‘‘Burlo Garofolo”, Trieste, Italy on the 18^th^ of January 2011 (Mariani Foundation grant ReMus R-11-85, Clinical Trial ID: NCT02316873; the trial registration was delayed for administrative reasons). The recruitment started the1^st^of March 2011 and finished the 9^th^of September 2011. Test phase: September 1 to October 11, 2011. Rehabilitation phase: October 17, 2011 to June 12, 2012. Retest phase: June 13, 2012 to August 2, 2012. Children participated only upon formal signed informed consent from their parents.

**Fig 1:**
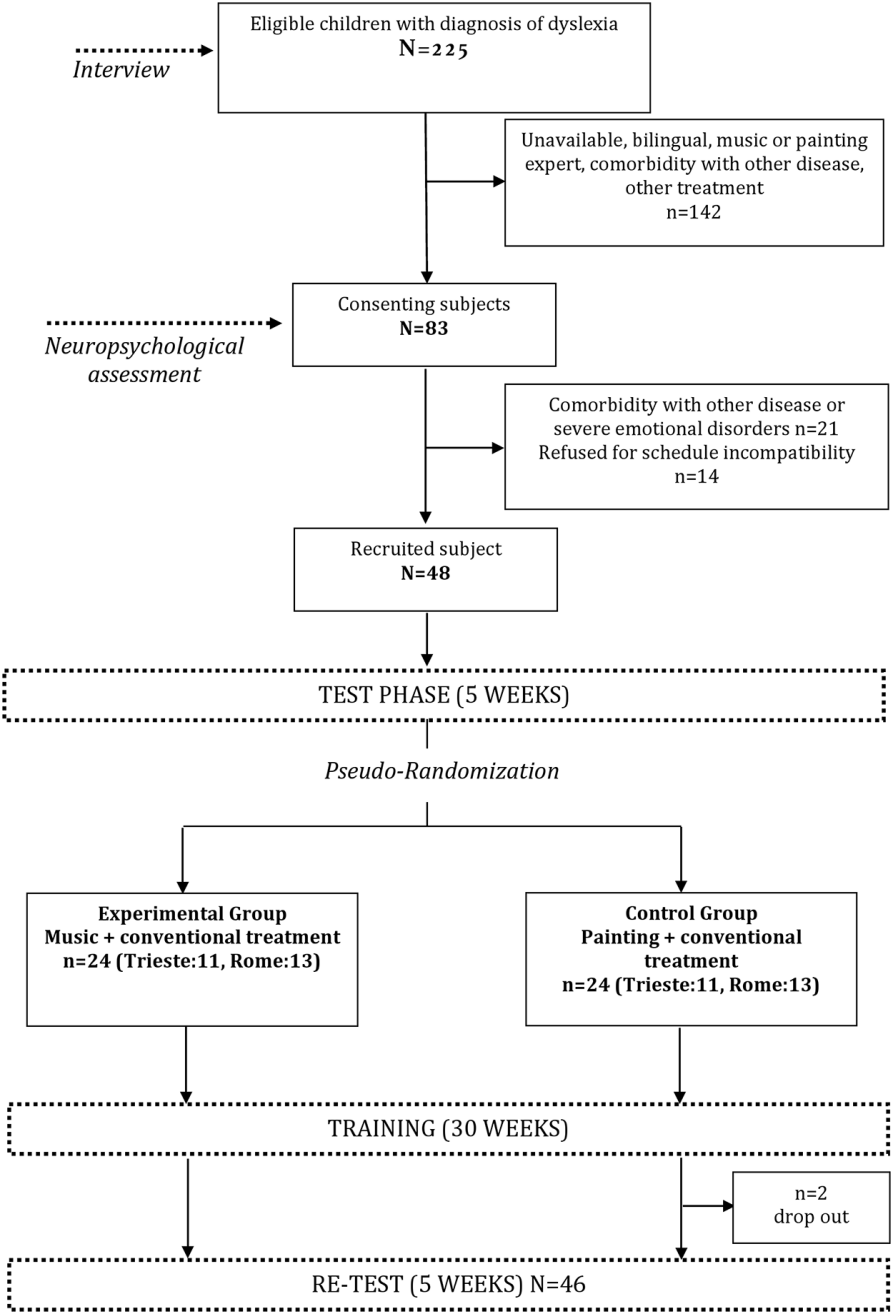
Flow chart illustrating participants’ recruitment and experimental design.

The eligibility of 225 children with a diagnosis of dyslexia (see Fig 1) was ascertained through anamnestic interview and neuropsychological assessment, on the basis of following criteria:

Inclusion criteria: Italian native language, reading performance (accuracy and/or speed) failed on at least two of three standardized Italian tests for school grade: text, words, pseudowords (cut-offs: z-score <-1.8 standard deviations from the mean for speed scores, a score <5th percentile in the accuracy scores), hearing and neurological examination within normal range, normal or corrected-to-normal visual acuity, General IQ >85 at the Wechsler Intelligence Scale for Children III.

Exclusion criteria: The selection was assessed in two steps. First, waiting lists and pre-existing clinical files were inspected to discard comorbidity. Diagnosis was made on the basis of the International Statistical Classification of Diseases and Related Health Problems 10th Revision (ICD-10) Version for 2010. More precisely, on the basis of ICD-10, we excluded all cases with the following disorders: Pervasive developmental disorders (F84), Neurotic, stress-related and somatoform disorders (F40-F48), Specific developmental disorders of speech and language (F80), Specific disorder of arithmetical skills (F 81.2), Mixed disorder of scholastic skills (F81.3), Other developmental disorders of scholastic skills (F 81.8), Developmental disorder of scholastic skills, unspecified (F81.9), Hyperkinetic disorders (F90), Conducts disorders (F91), Mixed disorders of conduct and emotions (F92), Emotional disorders with onset specific to childhood (F93), Disorders of social functioning with onset specific to childhood and adolescence (F94), Tic disorders (F95), and other behavioural and emotional disorders with onset usually occurring in childhood and adolescence (F98).

Second, during the first interview, Attentional Deficit Disorders with Hyperactivity (ADHD), Oppositional Defiant Disorder (ODD) and Conduct Disorder (CD) were assessed using the SNAP-IV (SNAP-IV Teacher and Parent Rating Scale, James M. Swanson, Ph.D., University of California, Irvine, CA 92715, in the Italian version) and children who scored over the cut-off were discarded [39]. Comorbidities with SLI were excluded on the basis of the clinical anamnesis, and, whenever necessary, on the basis of the Italian version of the TROG 2—Test for Reception of Grammar and the Italian version of the Peabody Picture Vocabulary Test.

#### Sample Size

On the basis of previous piloting on a smaller but similar sample, hypothesizing an average rate of reading improvement in a list of pseudo-words equal to 0,15 in the control group and of 0,25 in the experimental group (sd = 0,13), and considering α error = 5% and 80% power, 29 children needed to be recruited in each group for the study (two-tailed Mann-Whitney test).

Of the 62 eligible children, 48 children could join the program (22 in Trieste and 26 in Grottaferrata, Rome). Two children did not complete the program.

### Randomization Procedure

The study was a prospective, multicenter, open randomized controlled trial, consisting of test/rehabilitation/re-test. Children were pseudo-randomly assigned to two training groups—music and painting—on the basis of a baseline assessment in such a way that they did not significantly differ in any of the assessed dependent variables before training. These included neuropsychological tests, specifically-devised musical tasks (see below for a description of the tests) as well as information about sex, age, previous musical or painting experience(< = 12 months) and socioeconomic background (see Table 1).

**Table 1:** General description of the two groups.

	Painting (n = 22)	Music (n = 24)	p-value	test
**Male**	77%	71%	0,7	Fisher
**Age in years, mean (sd)**	10 (1)	10 (1)	0,8	Mann-Whitney
**Right handpreference**	86%	92%	0,8	Fisher
**School age, median (IQR)**	5 (4–7)	5 (4–6)	0,24	Mann-Whitney
**Music practice<1 year**	18%	21%	1	Fisher
**Painting practice<1 year**	0%	4%	1	1 Fisher
**Mother education level, mean (sd) 3 is for high school)**	3 (0,69)	2,83 (0,70)	0,64	Mann-Whitney

A p-value (two-sided) maximization algorithm was implemented on Matlab to minimize the differences between participants in the two groups in the tested variables (p always > 0.5 using Mann-Whitney for ordinal variables and Fisher exact for categorical variables).

One group was assigned to music classes and the other to painting classes for 2 hours/week over a period of 7 months. Children in both groups received a “conventional” rehabilitation program, carried out daily at home (see below Intervention). All children were retested after training, in a subset of the baseline tests. The children and the parents were blind as to which treatment was hypothesized to be more effective. The assessment was done by neuropsychologists and neurologists. While the clinicians were not blind to the treatment, they were blind during data analysis procedure, because the recorded material was coded.

### Neuropsychological Measures

In order to evaluate linguistic, musical, reading and general cognitive abilities, children were administered the tests reported below. Moreover, parents filled a detailed anamnestic module providing information about children’s health, including prenatal health, past illnesses and relevant family history.

#### General cognitive abilities

General cognitive abilities and working memory were evaluated through the Wechsler Intelligence Scale for Children III. After training, only a subset was used (see below Table 2).

**Table 2:** Summary of reading and phonological awareness results before and after training.

			Before training		After training			
Test	Outcomes Variables	Measure	Painting Group n = 24	Music Group n = 22	Painting Group n = 24	Music Group n = 22	Larger effect of music training	Effect size
**DDE-2 Pseudo word reading test**	**Accuracy (Primary Outcome)**	N of severely impaired children (<5)	15	17	12	5	**0.016**	OR = 3.7
	**Time**	Mean of z score of seconds	4.1 (3.0)	3.6 (2.6)	2.89 (2.6)	2.42 (2.2)	0.57 (***)	
**MT Text reading**	**Accuracy**	N of severely impaired children (<15)	21	20	15	8	**0.038**	OR = 1.9
	**Speed**	Mean of z score syill/s	-2.15 (0.84)	-1.83 (0.62)	-1.88 (0.81)	-1.68 (0.61)	0.51 (**)	
**DDE-2 Word reading test**	**Accuracy**	N of severely impaired children (<5)	18	15	8	7	0,76	
	**Time**	Mean of z score of seconds	6.2 (5.5)	5.6 (4.4)	4.18 (4.7)	3.38 (2.9)	0.67 (***)	
**Pseudo-word reproduction Promea Battery**	**Accuracy (40 items)**	Mean of correct Pseudo-words	32.45 (4.95)	31.33 (3.71)	33.77 (4.3)	34.87 (2.6)	**0.03**	0.3
**Phonemic segmentation task**	**Accuracy (38 items)**	Mean of correct words	13.5 (9.48)	17.1 (9.50)	20.36 (8.78)	23.52 (7.72)	0.7 (***)	
	**Speed**	Mean of seconds	437 (186)	429 (132)	401 (123)	397 (94)	0.17	
**Phonemic blending**	**Accuracy (38 items)**	Mean of correct words	9.4 (9.45)	11 (10.96)	14.05 (9.38)	19.83 (9.54)	**0.004**	0.4
	**Speed**	Mean of seconds	614 (153)	630 (153)	620 (207)	557 (148)	0.10	

The column “Larger effect of music training” reports whether or not there is a larger improvement in the music training group compared to the painting group (p value<0.05). When this is not the case (ns, p>0.05), significant main effects of session are reported (* = p<0.05; ** = p<0.01; *** = p<0.001), pointing to an equal improvement of both groups. The standard deviation from the mean is reported in parenthesis. The effect size is reported for significant effects (categorical data: Odd Ratio; interval data: z/√N).

*Auditory attention*: Auditory Attention was assessed using a test from the BIA Battery [40].

#### Phonological awareness

Phonological awareness was measured using pseudo-words repetition test of the Promea Battery [41].

#### Reading abilities

The ability to read aloud a text was assessed using an Italian standardized test for reading abilities MT Reading Test [42]. Since the test requires the use of different school-level adapted texts at the beginning and the end of each grade, which corresponded to our pre-test and test phase, statistical comparison was based on the standardized clinical cut-off. The ability to read aloud single words and pseudowords was measured on a standardized list of 102 Italian words and 48 Italian pseudowords words (*DDE-2)*[43].

#### Self-esteem

General competence self-esteem was evaluated using the corresponding subscale “Multidimensional test of self-esteem-TMA” [44].

#### Phonological awareness-Phonemic blending

The phonemic blending task comprised 38 words (nouns) of increasing difficulty, selected from VARLESS Italian database [45]. Children had to blend sounds into words (e.g. hearing [c]-[a]-[n]-[e] and producing [cane], (dog)). The number of correct items and time to perform the task were the dependent variables.

#### Phonological awareness—Phonemic segmentation

The phonemic segmentation task used 38 words, selected on the same criteria of the phonemic blending task. Children had to segment every word into its basic sounds (e.g. hearing [cane] and producing [c]-[a]-[n]-[e] (d-o-g).

#### Maximum likelihood Procedure—MLP rise time

In this task, children listened to a sequence of three identical pure tones (1 kHz with an onset and offset ramp of 10ms). Each tone lasted 800ms and the rise time of one of the three tones (randomized across trials) was varied adaptively (by lengthening the initial ramp). The first trial was set to a 25% difference (i.e. ramp duration of 200ms). Then, a Maximum Likelihood Procedure was used to find the subject threshold (MLP) [46]. Children had to detect the longest tone.

#### Maximum likelihood Procedure—MLP temporal anisochrony

In this task, children listened to a sequence of five identical equidistant complex tones. Each tone lasted 100ms and was followed by a 150ms gap. The gap separating the fourth tone from the previous and following tones was lenghtened adaptively to find the subject threshold using MLP. Children had to detect whether there was an irregular “jump” or not (regular foils were present to detect false positive).

#### Tapping

Children had to tap along a 90 pulse/minute metronome for 40 seconds. Each sound lasted 50 ms, was built using a sinusoidal sound (f = 1200), and ramped with a 1ms ramp at the onset and offset. Children listened to the metronome using an open headphone at approximately 75 dB and performed the task holding a pencil in their dominant hand and tapping it on a wooden box containing a microphone. They were instructed to tap as regularly as possible and did a short training before the recording to verify that they understood the task. Stimulation and acquisition were run using Audacity 1.3. Tap onsets were calculated using a custom Matlab program and a semi-automatic (supervised) procedure. Analyses were run on the coefficient of variation (i.e. the mean of the intertapping intervals divided by the standard deviation).

#### Rhythm reproduction

Children had to listen and reproduce 10 different rhythms (3 to 8 tones each; durations spanned from triplets of eighth notes to half notes, tempo was also set to 90 beats/minute). Each sound of the sequence lasted 65 ms and was built using a MIDI woodblock sound (f0 = 976 Hz). The same apparatus and settings described for the tapping task was used to deliver stimuli and record behavior. Every item performance was scored by two independent judges from 1 to 9 depending on its similarity to the template stimulus (1 no reproduction, 2 random order and random number of taps, 3 less number of beats, 4 correct number of taps but wrong temporal order, 5 rhythmic pattern contains one or two inter-beat interval errors, 6 rhythmic pattern contains one small error on a single inter-beat interval, 7 good pattern but "unstable" performance, 8 very good performance containing very minor rhythmic deviations, 9 perfect performance). The final mark for each child was the average of the twenty scores (inter judge correlation was 0.89).

#### Perception of musical meter

The musical meter task (same-different judgement) was adapted from Huss et al [31]. In the “different” trials (= 9), the change in metrical structure (beat = 500ms) was caused by adding 100 ms to the accented notes (also altering the beat interval; this seems to be a critical duration for children with developmental dyslexia in [31]).

More details on the tests can be found in Flaugnacco et al. [33].

### Outcomes

The primary outcome variable was the performance in the pseudoword reading test measured in terms of accuracy (percentile of number of errors).

The secondary outcomes variables were performance in:

- pseudoword reading test measured in terms of speed (z score of reading seconds);
- reading words and text, measured in terms of accuracy (percentile of number of errors) and speed (respectively z score of reading seconds and z score of syllables per sec);
- phonological knowledge, in particular in phonemic blending task (number of correct answers);
- auditory attention (number of correct answers);
- verbal short term memory and working memory, respectively in digit span forward and backward and in WISCIII QI Freedom from Distractability index, (raw scores and scaled scores);
- temporal processing through rhythm reproduction task, (raw score), tapping task (coefficient of variations) and temporal anisochrony threshold (ms).

### Intervention

Training sessions were carried out for seven months. Children in both groups received a “conventional” rehabilitation program, carried out daily at home for 20 minutes, with parents’ supervision. Every week, a series of activity forms containing exercises to be completed were given to each child. The activity forms were selected from training books/programs. In the Italian public health structures, this is the conventional treatment for children waiting for institutional rehabilitation, which often has a long waiting list.

#### Music training

This program was based on the Kodaly and Orff pedagogy and adapted to focus on rhythm and temporal processing (e.g. use of percussive instruments, use of rhythm syllables [ta, ti-ti, …], rhythmic body movements accompanying music, sensorimotor synchronization games). Two teachers professionally trained in music, with expertise in child pedagogy, were specifically selected and hired to perform the activity for this project. To avoid educational bias both teachers had to attend an intensive workshop on Kodaly Method, held by a Hungarian professor (Prof. Tamás Endre Tóth), formally trained at the International Institute “Z. Kodály” in Kecskemét. A common program was implemented.

#### Painting training

This program emphasized visual-spatial and hand skills as well as creativity. Two teachers professionally trained in painting, with expertise in child pedagogy, were specifically selected and hired to perform the activity for this project. Again to avoid educational bias both teachers had to attend an intensive workshop on Bruno Munari concepts of artistic education, held by an Art professor.

The activities and the materials to be used in both music and painting training were selected during the workshops and were continuously updated and shared by the teachers of the two centres. Training sessions involved groups of 5–6 children, one hour, twice a week, for 30 weeks (excluding holidays). Teachers were supervised by a neuropsychologist during the whole project. Teachers of both the music and art classes were told that an improvement was expected following both training types and that we expected each training type to favor specific competences.

### Statistical analyses

Non parametric testing was used to robustly assess whether or not:

1. Performance before training differed significantly between the two groups. Due to the pseudo-randomization procedure this was never the case in any of the variables of interest (Mann—Whitney U test).
2. Performance differed before and after training (Wilcoxon signed-rank test). This test assesses the global effect of session that comprises effect of the conventional intervention, effect of training, effect of retesting as well as effect of age maturation.
3. Changes in performance before and after training differed between the music and painting groups. This was the most relevant test because it assesses whether 1) music training has a significant effect on the variables of interest and 2) this effect is greater compared to the improvement that may be found in the control group [Mann—Whitney U test: (Music Group after training—Music Group before training) versus (Painting Group after training—Painting Group before training)].

Fisher's exact test was used when assessing the frequency distribution (MT test, reading words and pseudowords).

To assess whether the accuracy in the phonemic blending and segmentation tasks was influenced by working memory, rise time, temporal anisochrony, tapping and rhythm reproduction, two stepwise multiple regression analyses were performed after ensuring normality of the data (using Kolmogorov-Smirnoff and Lilliefors tests; the tapping variable had to be transformed into z-scores). Because the aim of the multiple regression was to show which was the best predictor of phonological awareness, both analyses (phonemic blending and segmentation) were run collapsing the two groups to maximize the sample size. In order to control for high multicollinearity problems we only used the measures after training. This also maximized the sample variance in the dependent variables. Statistical analysis were computed using Stata and Statistica.

## Results

### Pre-intervention Equivalence of Groups

The two training groups (music or painting) did not significantly differ in any of the assessed dependent variables before training (Table 1).

### Effects of intervention on outcomes variables

Children in both groups did not differ in terms of attendance and punctuality (median attendance = 47 and 46, p = 0.38; median punctuality = 3 and 2 delays for the music and painting group respectively, p = 0.21). There was also no group difference in terms of the appreciation of the training activities and the conventional treatment that was carried out daily at home for 20 minutes(positive appreciation for the activities: 96% and 90%,for the music and painting group respectively, p = 0.59; positive appreciation for the treatment = 42% and 38%, for the music and painting group respectively, p = 0.9).The outcomes in the reading and phonological tasks before and after training, both in terms of accuracy and speed, are reported on Table 2.

**Fig 2:**
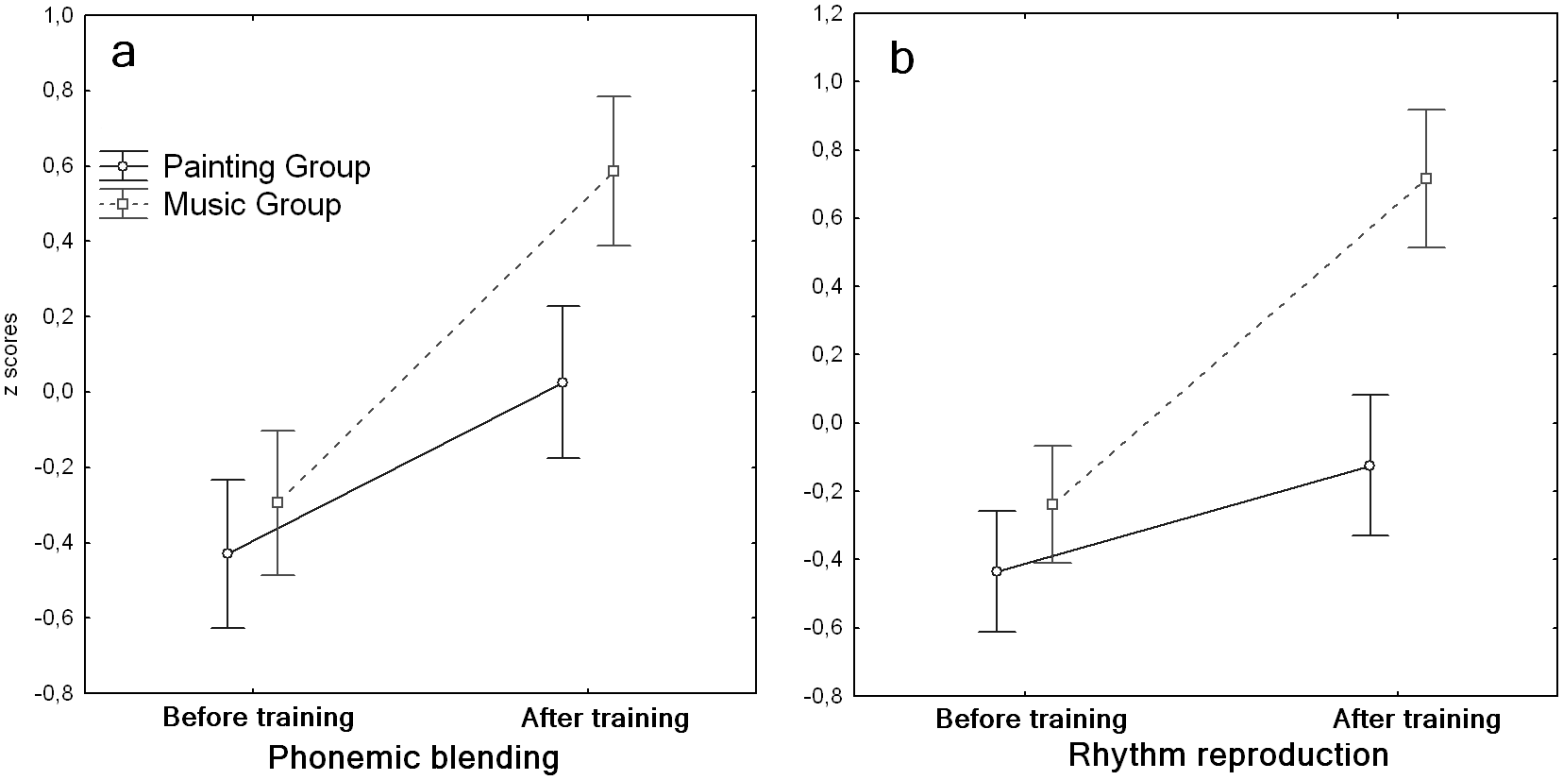
Effects of music and painting training on accuracy in the phonemic blending (a) and rhythm reproduction (b) tasks, before and after training. Error bars indicate the standard error of the mean. Values are z-score normalized.

As expected, the global outcome in the reading tasks after training showed a clear improvement in several measures in both groups. However, and most importantly, the music group outperformed the painting group in several reading tasks after training. In the text reading test, the proportion of children who were still considered as very poor performers was 50% less in the music group compared to the painting group. Similar results showing a greater improvement of the music group were obtained when measuring the accuracy in reading pseudo-words. No group differences were visible when measuring reading speed. Similarly to reading abilities, also phonological abilities globally improved. However, after training, the music group performed better than the painting group both in repeating Italian pseudo-words and in a phonemic blending task (blending sounds into words, e.g. hearing [c]-[a]-[n]-[e] and producing [cane] (dog), Fig 2a). Children in the music group also showed a greater improvement of working memory, as measured by the Wechsler intelligence scale (Table 3).

**Table 3:** Summary of results before and after training in WISC III Subtests, Auditory attention test, Self Esteem Competence Scale and Musical tasks.

		Before training		After training			
Test	Measure	Painting Group n = 24	Music Group n = 22	Painting Group n = 24	Music Group n = 22	Larger effect of music training	Effect size
**WISC III QI FD factor**	Mean of Composite Score	87 (12)	81 (12)	85 (18)	84 (13)	0.07	0.25
**WISC III Digit span**	Mean of Scaled Scores	8 (2.6)	7 (2.1)	7 (3)	8 (3)	**0.01**	0.35
**Digit span Forward**	Mean of Span Raw Score	4.1 (1.0)	4.2 (0.6)	4 (1.0)	4.2 (0.8)	0.4	
**Digit span Backward**	Mean of Span Raw Score	2.5 (0.7)	2.3 (0.5)	2.4 (0.8)	2.9 (1)	**0.02**	0.32
**Arithmetics**	Mean of Scaled Scores	8 (3)	7 (2)	8 (4)	7 (3)	0.9	
**Block design**	Mean of Scaled Scores	11 (3)	12 (3)	12 (3)	12 (3)	**0.02+**	-0.32
**Picture arrangement**	Mean of Scaled Scores	12 (2)	11 (2)	13 (3)	14 (2)	0.16	
**Vocabulary**	Mean of Scaled Scores	9 (3)	8 (2)	9 (3)	8 (3)	0.9	
**Similarities**	Mean of Scaled Scores	10 (3)	10 (2)	11 (4)	10 (3)	0.4	
**Self esteem scale**	Mean of Standard Scores	93 (12)	88 (10)	96 (10)	96 (11)	0.28*	
**Auditory attention Test**	Correct answers, Mean of raw score	8.4 (1.7)	7.7 (2.5)	7.7 (1.5)	8.8 (1.4)	**0.0037**	0.4
**MLP Temporal anisochrony**	Mean Threshold in ms	44,21	46,57	45,64	31,39	**0.0013**	0.45
**MLP Rise time**	Mean Threshold in ms	83,30	83,30	68,16	63,14	0.5*	
**Rhythm reproduction**	Mean accuracy	5	5	5	6	**0.0018**	0.45
**Metrical perception**	Mean accuracy	10,6	10,5	11,4	11,3	0.8	
**Tapping**	Coefficient of variation	0,11	0,10	0,08	0,08	0.9 ***	

The column “Larger effect of music training” reports whether or not there is a larger and significant improvement in the music training group compared to the painting group (p value<0.05), except in the Block design test wherein the improvement is larger for the Painting group (+). When this is not the case (ns, p>0.05), significant main effects of session are reported (* = p<0.05; ** = p<0.01; *** = p<0.001), pointing to an equal improvement of both groups. The standard deviation from the mean is reported in parenthesis.

This result was mostly driven by the inverse digit span test, which heavily relies on the phonological loop of the working memory system. By contrast, the painting group showed a greater improvement in perceptual visuo-spatial reasoning (assessed by the Block Design test). No differences were visible at the level of verbal comprehension or at the self esteem level, showing that group differences were not a mere greater motivational effect of music, but rather specific effects.

**Fig 3:**
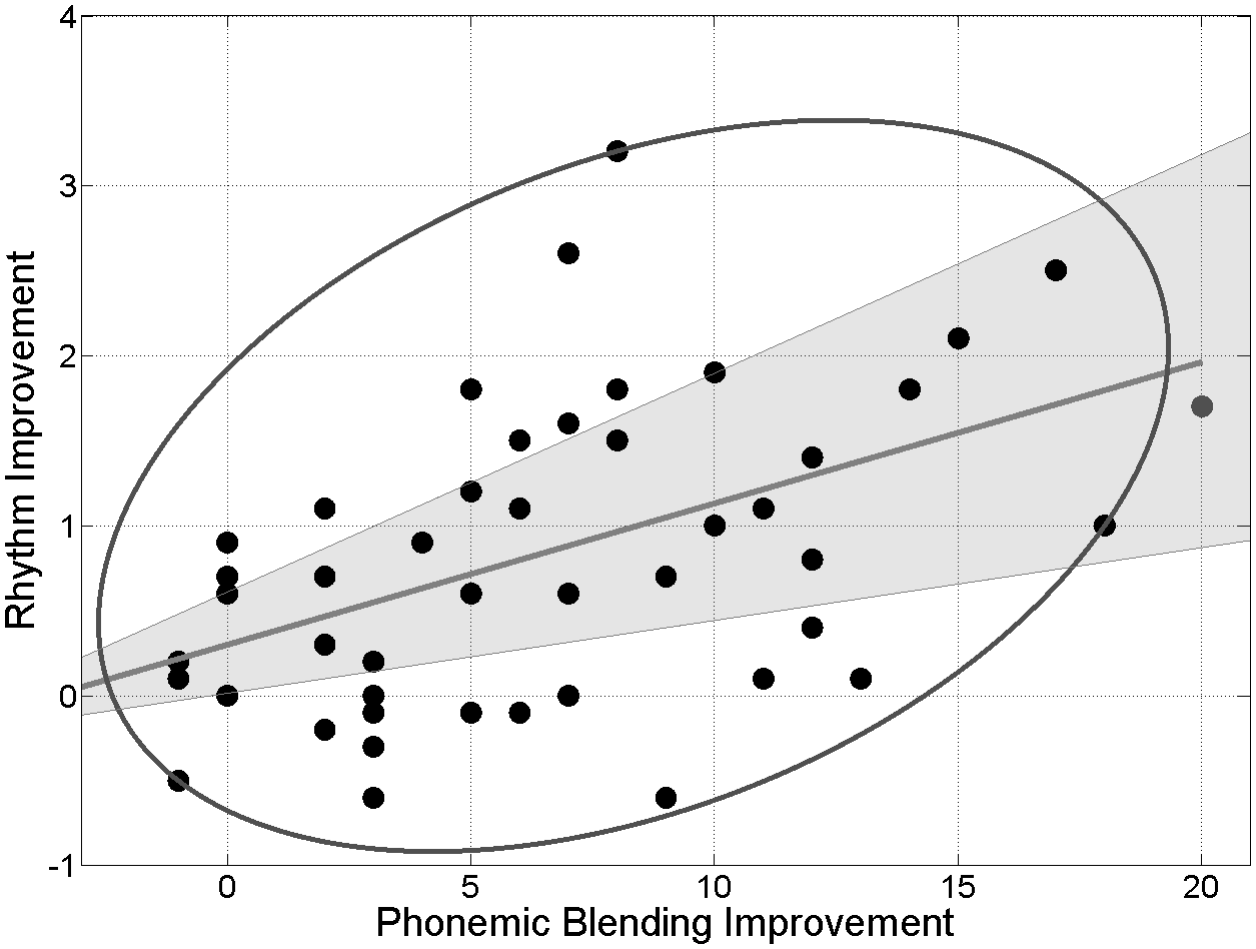
Scatter plot of the improvement in the rhythm reproduction task (accuracy After training—accuracy Before training) and the improvement in the Phonemic blending task (accuracy After training—accuracy Before training). The ellipse contains the non-outlying data. The grey line represents the best linear fit to the remaining data (Spearman skipped correlation, [47]). The shadow represents the 95% bootstrap CI.

A larger effect of music training was also found when testing auditory attention and in several perception and production abilities as tested by psychoacoustic and musical tasks (Table 3). This was particularly evident for tasks requiring precise temporal processing, such as the temporal anisochrony detection task (a psychophysical measure of temporal regularity perception) and the rhythm reproduction task (Fig 2b), wherein children had to tap a previously heard rhythmic sequence. The outcome in the rhythm production task turned out to be the best predictor of phonological awareness as measured by the phonemic blending and phonemic segmentation tasks (p<0.001 for both variables, betas = 0.56 and 0.48, respectively). Also the greater the improvement in rhythmic abilities, the greater the improvement in phonological awareness, as measured by the phonemic blending task (p = 0.002, r = 0.45, see Fig 3).

## Discussion

Our results show that music training can significantly improve reading skills in children with dyslexia. While reading speed improved in both groups, only the music group showed a clear improvement in reading accuracy. This result is important for two reasons. First, our sample consisted of 46 severe dyslexic children in absence of comorbid attention and/or language disorders—while indeed our sample was slightly smaller than what suggested on the basis of a pilot test, the power on the final data on the primary outcome was >0.80. Second, by contrast to other studies using a control group without any active engagement [36], the control group received an active training with a recreational component comparable to that of the intervention group. While the motivational effect of a musical activity compared to a control group without any active engagement is certainly high, our results show that children in the two groups did not differ in their appreciation of the activity or in their self-esteem after training. Thus, the transfer effects described here seem to be due to precise neural and cognitive mechanisms that are specifically enhanced by music training.

The improvement in reading was not limited to text reading but included pseudo-word reading, which is usually quite difficult to train [48] and often remains impaired in dyslexic adults [49]. Reading pseudo-words requires an active grapheme-phoneme conversion, which is a rather complex process. The development of phonological awareness follows a hierarchical progression, from larger to smaller phonological units: whole spoken words—syllables—onset rhymes—phoneme level units of language. The latter is called phonemic awareness and develops following introduction to reading and is affected by the variations in orthographic consistence among languages [2]. In Italian, phonemic blending relies more on phonological rather than lexical processing, the contrary of what one would expect in less transparent orthographies [50]. The improvement we observed in the phonemic blending task is thus in line with previous findings showing a tight link between phonological and musical competences [51] and a positive effect of music training on phonological abilities in both normal readers [26] and pre-schoolers [52]. Moreover, Kraus and collaborators recently showed that beat synchronization, precise neural encoding in the brainstem and language skills are highly intertwined in preschoolers [53].

Interestingly, the best predictor of phonological awareness was not working memory nor auditory attention but rhythmic reproduction. Even further, the improvement in rhythmic reproduction was a good predictor of the improvement in phonological abilities. This supports the hypothesis of a causal role of rhythm-based processing for language acquisition and phonological development [54]. Moreover, this can also be interpreted within the temporal sampling framework for developmental dyslexia, which has been proposed to account for auditory sensory difficulties and rhythmic impairments often found in children with dyslexia [8].

While we did not find a significant correlation between amplitude envelope processing and phonological abilities, as could be expected on the basis of previous correlational research [32], this was possibly due to a lack of sensitivity in some of our tasks. Indeed what is of interest here is not the global performance on a given task, that would be appropriate in a correlational approach, but the more specific improvement of the performance following music training and its relation to improved phonological abilities. For instance, the task measuring metrical perception, adapted from Huss and collaborators [31], did not show any improvement following music training (nor painting training), thus preventing any possible correlation with improved phonological abilities. Nonetheless, metrical structure is a major determinant of the perception of rhythm, which in turn was the best predictor of phonological awareness. Concerning tapping skills and rise time detection, while they did significantly improve after training, this improvement was not different across training groups. Thus, changes in performance are possibly due to maturation and repetition of the tasks. The finding that the rhythmic reproduction task is the best predictor of phonological abilities may be due to the fact that this task is possibly the most complete (and complex) temporal task that we assessed. Indeed, it requires to precisely process temporal anisochronies, temporal envelops (i.e. durations), beat and metrical structure, to store an auditory temporal representation of sounds as well as to have good sensorimotor skills allowing a precise reproduction of the internalized representations.

Another measure that was significantly affected by music training and that strongly correlated with phonological skills was a measure of temporal anisochrony, namely the ability to detect the temporal regularity of an auditory sequence. As described by the dynamic attending theory, rhythmic perception depends upon stimulus-neural coupling [55]. Moreover, entrainment can optimize processing resources when events take place at expected moments in time [56]. In speech, oscillatory neural mechanisms that entrain to the amplitude envelope of ongoing speech are also thought to serve to guide perception and affect sensitivity to relevant speech acoustic features [57]. Indeed, sound envelope modulations, the building block of speech rhythm, play a major role in speech comprehension in both adults and infants [58, 59]. Interestingly, the temporal sampling needed to process rhythm in music and syllables, words and prosody in speech is very much alike and is known to be impaired in persons with dyslexia [8, 28, 60]. These data and our findings support the idea of a tight and possibly causal [61] link between temporal perceptual accuracy and phonological skills [33, 53], although further work will be needed to disentangle the different levels of temporal processing and their respective links to speech and reading abilities.

Overall, we show that music training can have a positive causal effect on specific linguistic abilities that are impaired in dyslexic children. This seems to take place thanks to an improvement in rhythmical and metrical abilities. Training these skills most probably results in improved auditory processing, prosodic and phonemic sensitivity [62], sequencing abilities, auditory and temporal orienting of attention, and certainly more.

Importantly, these music training effects do not generalize to all tested competences. For instance vocabulary or mathematical skills did not improve more in the music group. Also, the painting group improved more than the music group in visuo-spatial abilities. Given that the playful and emotionally engaging nature of training certainly played a role, children in both groups equally attended and appreciated training sessions. Thus, music training seems to have a specific effect on those perceptual and cognitive abilities that are shared by music and language.

Our findings strongly support the hypothesis of a beneficial effect of music training on reading skills and phonological awareness. They also highlight an important role of rhythm on phonological perception and production. Since rhythm and meter, by requiring more precise timing, possibly place higher demands in music than in language [28], remediation based on music and rhythm may strengthen phonological and language development from a perspective that is quite different from (though complementary to) the more traditional language-based remediation approaches.

Through the enhancement of rhythmic skills and by means of its emotionally engaging and joyful nature, music might also become an important tool for early classroom interventions for children who are at risk of dyslexia.

## Supporting Information

S1 CONSORT Checklist CONSORT 2010 checklist for a randomised trial. (DOC)

S1 File Ethical Approval. (PDF)

S2 File Research Grant Application. (PDF)

S1 Protocol Clinical Trial Registration Protocol. (PDF)
