# Supplementary material for: Music Training Increases Phonological Awareness and Reading Skills in Developmental Dyslexia: A Randomized Control Trial
Source: PLoS One. 2015 Sep 25;10(9):e0138715. doi: 10.1371/journal.pone.0138715 (PMC4583182; doi:10.1371/journal.pone.0138715)
Supplement: S1 File — (PDF) [file pone.0138715.s002.pdf]

BURLO

Comitato Indipendente per la Bioetica (C.I.B.)

Prot. 638 MM

Prot. L. – 1195

Trieste, 18 GEN. 2011

IRCCS "Burlo Garofolo" Trieste
SERVIZIO SPERIMENTAZIONI CLINICHE
18 GEN 2011
PROTOCOLLO N. 17/11

Egregi

Prof. G. Zauli
Direttore Scientifico

Dott.ssa S. Zoia
S.C. Neuropsichiatria Infantile

Sede

Oggetto: Progetto *"Diagnosis and Rehabilitation with music: instrument for the diagnosis and rehabilitation of neuropsychological disorders in children, by means of temporal and pitch processing of music – DYAREMUS"*

Si invia in allegato il parere etico relative allo studio di cui all'oggetto espresso dal Comitato Indipendente per la Bioetica nella seduta del 10 gennaio u.s.

Cordiali saluti

Edvige Gombach
Segreteria C.I.B.

E. Gombach

Comitato Indipendente per la Bioetica (C.I.B.)
Istituto di Ricovero e Cura a
Carattere Scientifico pediatrico
Burlo Garofolo
Ospedale di alta specializzazione e di rilievo nazionale per la salute della donna e del bambino

● 34137 Trieste ● Via dell'Istria 65/1 ● tel.+39.040.3785.340 ● fax +39.040.3785210 ● e-mail: comitato.bioetica@burlo.trieste.it ●

BURLO
Comitato Indipendente per la Bioetica (C.I.B.)
I.R.C.C.S. Burlo Garofolo
Via dell'Istria 65\1 34137 Trieste

Segreteria Scientifica
Tel 040 3785 340 Fax 040 3785 210

e-mail: comitato.bioetica@burlo.trieste.it

Prot. CE/V- 118

VERBALE della Riunione del 10 gennaio 2011

NOMINATIVO	QUALIFICA	PRESENTI
Dott. Furio Bouquet	Esperto di bioetica - Presidente	SI
Prof.ssa Flora Bartoli	Farmacologo - Università degli Studi di Trieste	SI
Prof. Luigi Cattin	Esperto di Ricerca Clinica – Università degli Studi di Trieste	SI
Dott.Massimiliano du Ban	Rappresentante associazionismo	SI
Dott. Dino Faraguna (sostituito dalla dott.ssa P. Visconti)	Direttore Sanitario - Pediatra	SI
Dr.ssa Giuseppina D'Ottavio	Ginecologo	SI
Dott.ssa Olivia Giannini	Rappresentante dei Medici di Medicina Generale	SI
Dott.ssa Giuliana Pitacco	Esperta infermieristico	SI
Dott.ssa Rossella Paparazzo	f.f. Direttore di Farmacia	SI
Dr. Luca Ronfani	Pediatra - Esperto di Biostatistica	SI
Dr. Angelo Venchiarutti	Esperto in materia giuridica	SI
Prof. Giorgio Zauli (sostituito dal dott. G.A. Zanazzo)	Direttore Scientifico	SI

Edvige Gombach	Segretaria	SI

Ordine del Giorno: Comunicazioni del presidente
Valutazione dei progetti di Ricerca e di protocolli di Sperimentazione pervenuti

... *Omissis* ...

- In data 21 dicembre 2010 è pervenuta la richiesta di parere etico per

Comitato Indipendente per la Bioetica (C.I.B.)
Istituto di Ricovero e Cura a Carattere Scientifico pediatrico
Burlo Garofolo
Ospedale di alta specializzazione e di rilievo nazionale per la salute della donna e del bambino
● 34137 Trieste ● Via dell'Istria 65/1 ● tel.+39.040.3785.340 ● fax +39.040.3785210 ● e-mail: comitato.bioetica@burlo.trieste.it ●

l'Emendamento n. 1 al Progetto ***"Diagnosis and Rehabilitation with music: instrument for the diagnosis and rehabilitation of neuropsychological disorders in children, by means of temporal and pitch processing of music – DYAREMUS"*** - Richiedente: dott.ssa S. Zoia.

Presa visione della documentazione presentata sia sotto il profilo scientifico che sotto quello etico, il Comitato esprime parere favorevole.

... *Omissis* ...

E. Gombach
Segreteria CIB

F. Bouquet
Presidente CIB

Comitato Indipendente per la Bioetica (C.I.B.)
Istituto di Ricovero e Cura a
Carattere Scientifico pediatrico
Burlo Garofolo
Ospedale di alta specializzazione e di rilievo
nazionale per la salute della donna e del bambino

● 34137 Trieste ● Via dell'Istria 65/1 ● tel.+39.040.3785.340 ● fax +39.040.3785210 ● e-mail: comitato.bioetica@burlo.trieste.it ●
