# Supplementary material for: Music Training Increases Phonological Awareness and Reading Skills in Developmental Dyslexia: A Randomized Control Trial
Source: PLoS One. 2015 Sep 25;10(9):e0138715. doi: 10.1371/journal.pone.0138715 (PMC4583182; doi:10.1371/journal.pone.0138715)
Supplement: S2 File — (PDF) [file pone.0138715.s003.pdf]

Research Grant Application Form

Biennium 2011-2012

The deadline for online submission is Wednesday June 30, 2010.

NOTES

The application form should be entirely filled out in **English**.

The Application Form (A.F.) is organized into **sections**, which should be **completed in any order**.

The A.F. and Privacy Statement must be submitted online. The Applicant is also required to send **by mail a hard copy with signatures** as well as a **copy on CD** to:

Fondazione Mariani, Viale Bianca Maria 28, 20129 Milano (please specify "Mariani Grants 2010")

1.

Title of the project / Titolo del progetto

Rhythm and music to rehabilitate reading disorders (ReMus)

2.

Principal investigator in charge of the project /
Responsabile Ricerca
ZOIA STEFANIA

Title / Qualifica
Psychologist

Department / Institute Divisione/ Istituto
Child Neurology and Psychiatry Ward, IRCCS Institute for Maternal and Child Health “Burlo Garofolo”

Address / Indirizzo
IRCCS “Burlo Garofolo”, SCO NPI, Via dell’Istria, 65/1 - 34137 - Trieste (Italy).

Telephone / Telefono
++39 040-3785405

Fax

++39 040 3785544

e-mail: zoia@burlo.trieste.it

% effort on the project
% del tempo che intende dedicare alla ricerca
20%

3.

Collaborators from above mentioned Institute (please specify if full-term employees, recipients of boursaries, scholarships, fellowships, ecc. end % effort on project for each of them)
Collaboratori interni all'Istituto specificando la qualifica, se di ruolo, titolari di borse o altro e % del tempo che ciascuno intende dedicare alla presente ricerca

**Last Name First Name, title and details on the above**<br>**Cognome, Nome, qualifica e specifiche**	**% effort (time)**
Carrozzi Marco, MD, Child Neurologist, Director of Child Neurology and Psychiatry Ward full time	5
Skabar Aldo, MD, Child Neurologist, First Level Medical Manager, full-time	5
Lonciari Isabella, Psychologist, permanent position, full-time	10
Bravar Laura, Psychological tester, Permanent Position, full time	10
Ronfani Luca, MD, Paediatrician, researcher at the Epidemiology Unit, full time	10

4.

Collaborators from other Institutes (please specify the Institute and if full-term employees, recipients of boursaries, scholarships, fellowships, ecc. end % effort on project for each of them)
Collaboratori esterni, specificando l'Istituto, la qualifica, se di ruolo o titolari di borse o altro e % del tempo che ciascuno intende dedicare alla presente ricerca

**Last Name, First Name, Insitute, title and details on the above**<br>**Cognome, Nome, Istituto qualifica e specifiche**	**% effort (time)**
Lopez Luisa, MD, Specialization in Neurophysiopathology, PhD in Neuropsychopathology of learning processes in the developmental age. Director of Child Neurology and Psychiatry out-patient ward, Center "Eugenio Litta" (Grottaferrata, Rome), permanent, full time	20
Terribili Chiara, Consultant Psychologist and Cognitive and Behavioral Psychotherapist, Child Neurology Rehabilitation Center "Eugenio Litta" (Grottaferrata, Rome)	15
Schön Daniele, graduated in Experimental Psychology, PhD in Neurosciences, Cello Diploma. Researcher, full time, permanent position, Mediterranean Institute of Cognitive Neurosciences - CNRS Marseille (France)	20
Besson Mireille, PhD, Director of research, INCM, CNRS, Mediterranean Institute of Cognitive Neurosciences - CNRS Marseille (France)	4
Habib Michel, Timone Hospital & associated researcher to INCM, CNRS, Marseille MD, PhD, Neurologist, Director of ResoDys	4

5.

Total financing requested (maximum: 250.000 Euros)
Finanziamento complessivo richiesto (massimo 250.000 euro)
€ 139,663.00
Duration of the Project (maximum 2 years)
Durata del progetto (Massimo 2 anni)
2 years

---

6. Co-financing: please indicate current sources that contribute as complements to the Project:
Co-finanziamento, si prega di indicare le risorse se effettivamente complementari al progetto:

IRCCS Burlo Garofolo Trieste
*Personnel **€** 64,000.00

"Eugenio Litta" Grottaferrata
*Personnel **€** 28,000.00

INMC-CNRS Marseille
*Personnel **€** 34,000.00

*These funds must be considered as the monetary equivalent of the amount of time spent by the researchers for the project (tempo-uomo).

Budget and Duration of Co-financing
Importo e durata del co-finanziamento

Total Budget of Co-financing € 126,000.00

Duration of Co-financing: 2 years, until 31/12/2012

Overlap with the Mariani Foundation application, if any
Sovrapposizione con l'applicazione della Fondazione Mariani, se ci sono

---

7.

Summary of the project and detailed schedule - *maximum length: 4.000 characters*
Riassunto del progetto con il dettaglio dello svolgimento cronologico - *massimo 4.000 battute*

Background:
Poor performance in tasks requiring temporal processing, discrimination of acoustic variations (e.g. envelope and duration), rhythm perception and synchronization of movement to sound, seems to be a crucial factor underlying reading disorders (RD) in children. As speech, music may be considered a sequence of sounds temporally organized. Interestingly, children with RD show deficits in temporal processing, both in language and in music [16, 10, 5]. Recent advances in the rehabilitation of RD seem to demonstrate that musical activity increases phonological awareness, word segmentation, working memory, as well as reading abilities [9, 10, 13]. However, new research studies are necessary to investigate the efficacy of a musical intervention based on rhythm activities.

Objectives
The aim of this project is to test the hypothesis that musical training improves the development of phonological awareness and reading skills by improving temporal processing. Many authors [3, 8, 9, 16] have specifically underlined the need for controlled studies to address the idea that music training may improve language and reading skills. So, after a thorough clinical assessment, the objective is to propose a highly motivating, ecological and amusing intervention program, focused on rhythm and temporal processing.

Plan
Participants will include a minimum of 60 school-aged (8-12 yrs) children with RD, recruited at the Burlo Garofolo Institute (Trieste) and the Clinic of Child Neurology Rehabilitation Centre (Grottaferrata, Rome). Only children with a diagnosis of RD, without comorbidity involving Attentional Deficit disorders with Hyperactivity (ADHD), Specific Language Impairment (SLI) and Oppositional Defiant Disorder (ODD), will be included.
In addition to specific neuropsychological assessment with standardized tests, each child will also evaluated in tasks requiring melody, rhythm and timbre elaborations. Along with a conventional rehabilitation treatment, each participant will be pseudo-randomly assigned to either the musical training group (experimental group), for participation in a temporal processing (rhythm) programme, or a painting group (control group). The trainings will last 30 weeks excluding holidays, one hour twice weekly and will be carried on by two musicians and two art teachers, with expertise in child pedagogy. Training will be followed by both an immediate and delayed (6 months) re-testing sessions.

Methodology
The study is a prospective, multicentre, open randomized controlled trial, consisting of test/rehabilitation/re-test/follow-up. The assessment will include both standardized tests (eg. WISC III, Phonological awareness, Reading and spelling test, Movement ABC, etc.) and specifically devised musical tasks. Rehabilitation will consist in a conventional procedure for all children. Moreover, each child will be pseudo-randomly assigned to either an "experimental" training (Kodaly-Orff method, focused on temporal/rhythmical aspects) or a "control" training (painting). Re-testing and follow-up will allow comparing the impact of music or painting on performance, both on a short and a long term.

The musical training proposes to be an efficient rehabilitative method to improve spectral-temporal and rhythm processing. As reported in literature, musical intervention can influence the evolution of learning abilities in children, with immediate positive transfer effects on the cognitive and emotional development, with long lasting effects on academic achievement. We intend to improve accuracy in phonological awareness, digit span, reading and spelling abilities and rapidity (syllables/sec) in reading skills. This

project may lead to identify a new rehabilitation treatment for children with reading disorder. Results will be published in international peer-reviewed journals.

8.

Scientific Background - ***maximum length: 2.000 characters***
Background scientifico - *massimo 2.000 battute*

Musical expertise has been shown to improve several aspects of auditory processing as well as language and literacy skills [2, 3, 9, 10, 15, 12]. In particular:

- music perception ability is predictive of reading skills [2];
- musical perceptual abilities correlate with phonological awareness, reading abilities [2] and second language learning [15];
- musical training improves reading abilities in typically developing children [9];
- musical activity increases memory, as well as attention and language abilities in school-aged children (6-11yrs.) [9, 7, 3, 12, 13].

Children with reading disorders often show impairments in several domains of temporal processing. The strategies used by these children in reproducing rhythm or following the pace of a metronome indicate deficits in analysing temporal structures, sequencing and synchronising sounds and actions [19].
Although there are several theories on the causes of RD [11, 20], many authors agree that the phonological deficit in reading disorder is based primarily on a low level auditory dysfunction associated to temporal processing [7, 8, 14, 16].
This project focuses on the rehabilitation of children with RD by improving rhythm perception and production as well as spectral processing which should in turn increment the phonological awareness skills necessary for reading.
The scientific background of this project is based on two lines of research, one underlining the difficulties in the perception of the formant transition duration in children with specific language impairment [16], and the other a deficit in rise-time of sound envelope (the variation in sound amplitude through time) in children with specific language impairment and RD [5, 7].
Therefore a treatment directed to low-level processing (such as auditory discrimination and temporal processing) will be offered in order to improve high-level processing, with less effort for the child.

9.

Research Plan (objectives, methods, preliminary results and essential references). Please specify the innovative aspects of the project, its time-frame organization, and verifiability of results - ***maximum length: 7.000 characters***
Progetto di Ricerca (obiettivi, metodologie, risultati attesi e referenze essenziali) - Specificare in particolare gli aspetti innovativi del progetto, le fasi operative e la verificabilità dei risultati - *massimo 7.000 battute*

OBJECTIVES

The aim of this study is to test the hypothesis that musical training will improve the development of reading skills and phonological awareness by improving temporal processing. While this hypothesis is emerging in contemporary research literature, it has not been directly tested by focusing on temporal aspects. Experts in reading disorders have specifically indicated the need for controlled studies to address the idea that music training may improve reading and language skills [3, 10, 7, 17]. The study will involve children with RD who do not have equal phonological impairment, since RD can also be caused by visual-deficits and/or multisensory spatial attention problems [6]. In keeping with the above, we intend to apply a longitudinal approach to evaluate the effects of a highly motivating intervention program, based on fast (spectral) and slow (rhythmic) temporal processing. We believe the results will prove very useful in developing new, efficient and amusing rehabilitation and education programs for children with reading impairments.

METHODS

The study is a prospective, multicentre, open randomized controlled trial.

Participants

Inclusion criteria: Italian native language children with RD [1], hearing and neurological examination within normal range, WISC III IQ>85, diagnosed and recruited at the "IRCSS Burlo Garofolo" (Trieste) and the Institute "Eugenio Litta" (Grottaferrata).
Exclusion criteria: presence of comorbidity involving Attentional Deficit Disorders with Hyperactivity (ADHD), Specific Language Impairment (SLI) and Oppositional Defiant Disorder (ODD); previous formal musical or painting education (fig. 1).
Children will participate only upon formal signed informed consent from their parents.
We will recruit a minimum of 60 children, aged 8 to 12 years, 30 in Trieste and 30 in Grottaferrata.
Children recruitment will be facilitated by the fact that the 2 institutes have a large *pool* of children with learning disorders.

Procedure

After checking the inclusion and exclusion criteria, children will be pseudo-randomized to (fig. 1; table 1):

1) "experimental group" (music-training). Musical activities, based on the Kodaly-Orff Method, will be adapted to focus on fast (spectral) and slow (rhythmic) temporal processing. Children will be trained to discriminate and produce different sounds (timbres) and rhythms using several instruments as well as by singing. By reinforcing the link between the sound and the action producing it, children will not only improve their musical skills, but also more general auditory and temporal processing abilities.
2) "control group" (painting-training). The activities will emphasize visual-spatial and hand skills. The children will work on the fundamentals of painting (colour, light, texture, lines and perspective), as well as on their manual skills

The music or painting activity will be organized for groups of 5 children, one hour twice weekly, for 30 weeks excluding holidays. It will be carried on by two musicians and two art teachers, with expertise in child pedagogy, supervised by psychologists.
Children in both groups will receive a "conventional training" program to be carried out daily at home. Since waiting lists for treatment of children with RD are usually lengthy (even 8 to 12 months), the first intervention often takes place at home. This research will adopt the same modalities: parents will be provided with didactic materials (table 4) to be used under the supervision of psychologists and MDs.
The pseudo-random assignment to either music or painting group, following the first testing phase, will take care of balancing main cognitive and social variables across groups.
Hypothesizing an average ratio of reading errors in the list of pseudo-words equal to 0,25 (SD of 0,15) in the control group [3] and of 0,15 in the experimental group, and considering α error = 5% and 80% power, at least 60 children need to be recruited for the study.

Materials

General Assessment section
In order to evaluate cognitive, linguistic, academic, attentive, motor and musical abilities children will be administered all the tests reported in tables 3-4. Moreover, parents will be requested to fill in a detailed anamnestic module to provide information about their child's health, including their prenatal health, past illnesses and family history for the disorders of interest in this research. A section of the questionnaire will also consider the family's social-cultural background (Table 2).

Experimental tasks section
Musical tasks will include threshold estimation for pitch/duration/rise-time/temporal synchrony as well as melody and rhythm discrimination and memory tasks. Several production tasks will also be used, including singing and tapping hands (Table 3).
Children will be tested individually twice during a 2 hours session. Tests will be administered before randomization (baseline), immediately after training and after six months (Tables 1, 5).

Data Analysis

The two groups will be compared by their main baseline characteristics to evaluate the efficacy of the randomization. To evaluate differences in the study outcomes between experimental and control groups, analyses of variance will be used for continuous variables and chi-square test for categorical variables.

Multivariate analyses will be carried out to take into account potential influencing factors such as age, sex, perinatal suffering and other variables related to health and socio-cultural background.

References

1. American Psychiatric Association. (DSM-IV-TR). Washington, 2000.
2. Anvari, S. H. et al. (2002). J. of Experimental Child Psychology, 83:111-130.
3. Besson, M. et al. (2007). Restorative Neurology and Neuroscience, 25: 1-12.
4. Campanini, S. et al. (2010). Dislessia, 7 (2): 165-179.
5. Corriveau, K. et al. (2007). Journal of Speech, Language and Hearing Research, 50: 647–666.
6. Facoetti, A. et al. (2009). Journal of cognitive neuroscience, doi:10.1162/jocn.2009.21232.
7. Goswami, U. et al. (2010). Developmental Science, DOI: 10.1111/j.1467-7687.2010.00955.x
8. Habib, M. (2000). Brain, 123: 2373-2399.
9. Moreno, S. et al. (2008). Cerebral Cortex, doi:10.1093/cercor/bhn120.
10. Overy, K. (2003). Annals of the New York Academy of Sciences, 999: 497-505.
11. Ramus, F. (2004). TRENDS in Neurosciences, 27 (12): 720-726.
12. Schellenberg, E. G. (2004). Psychological Science, 15(8) : 511-514.
13. Schellenberg, E. G. et al. (2010). Psychology of Music, 10.1177/0305735609339473: 209–221.
14. Schulte-Körne G. et al. (2010) Clinical Neurophysiology, (28) doi:10.1016/j.clinph.2010.04.028.
15. Slevc, R. et al. (2006). Psychological Science, 17 (8): 675-681.
16. Tallal, P. (2000). In D.V.M. Bishop e L. B. Leonard. Philadelphia: Psychology Press.:131-155.
17. Tallal, P. et al. (2006). Trends in Neurosciences, 29 (7): 382-390.
18. Thomson, J.M. et al (2008). Journal of Physiology Paris, 102: 120–129.
19. Wolff, P.H. et al. (2002). Reading and Writing: an Interdisciplinary Journal, 15: 179-206.
20. Ziegler, J. C. (2006). Brain and Language, 98: 341–343.

10.

Expected Results and suggestive Measures of Outcome - *maximum length: 1.500 characters*
Risultati attesi e risultati indicativi - *massimo 1.500 battute*

In both groups (painting and music training), we expect a general improvement in performances on several tests, due to age, test repetition and motivation related to the playful activity proposed. However, since the rehabilitation treatment with music is specifically focused on temporal processing and rhythm, we predict that it will improve children's performance not only on musical but also on phonological and reading tests, by enhancing spectral-temporal perception and rhythmic production. The expected amelioration of musical and linguistic abilities, following the specific musical training, will be quantified and compared to the control group (painting) by searching significant differences on the crucial tests. The main variables will be the number of correct answers in phonological tests (CMF Test, cf. table 2) and the number of errors and speed in reading, measured as syllables/second (Word, pseudo-word and MT reading tests, cf. table 2). All these values are critical considering that music training will improve both fast (spectral) and slow temporal (rhythmic) auditory processing with a potential positive transfer to phonological and suprasegmental (prosodic) processing. Thus our project may allow to target an efficient and playful rehabilitation treatment for children with reading disorder.

11.

List of References of Principal Investigator - *maximum 10 relevant references*
Bibliografia del responsabile del progetto - *massimo 10 voci pertinenti*

1. Zoia, S., Blason, L., D’Ottavio G., Bulgheroni, M., Pezzetta, E., Scabar, A. & Castiello, U. (2007) Evidence of early development of action planning in the human fetus: A Kinematic study. Experimental Brain research, 176 (2): 217-226 (10).

2. Zoia, S, Barnett, A., Wilson, P. & Hill, E. (2006). Developmental Coordination Disorder: Current Issues. Child: Care, Health and Development, 32 (6).

3. Zoia, S., Pezzetta, E., Blason, L., Scabar, A., Carrozzi, M., Bulgheroni, M., Castiello U. (2006). A Comparison of the Reach-To-Grasp Movement between Children and Adults: A Kinematic Study. Developmental Neuropsychology. 30 (2): 719-738.

4. Zoia, S., Borean, M., Bravar, L. (2005). Handwriting in Italy. In A. Barnett (Ed.) Handwriting. The Handwriting Interest Group, Edding, UK, 4: 16-19.

5. Zoia, S., Castiello, U., Blason, L., Scabar, A. (2005). Reaching in Children with and without Developmental Motor Coordination Disorder under normal and perturbed vision. Developmental Neuropsychology. 25 (2): 257-273.

6. Zoia, S. (2005). Valutazione cognitiva nella prima infanzia. In S. Bonifacio & L. Hvastja Stefani (eds) L'interazione comunicativa e linguistica nel bambino con ritardo di linguaggio. Tirrenia, (Pisa): Ed. Del Cerro, 163-184.

7. Zoia, S. (2004) Lo sviluppo motorio nel bambino. Roma: Ed. Carocci.

8. Zoia, S., Pelamatti, G., Rumiati, R. (2004). Praxic skills in Down and Mentally Retarded adults: Evidence for multiple action routes. Brain and Cognition, 54: 7-17.

9. Zoia, S., Pelamatti, G., Cuttini, M., Casotto, V., Scabar, A. (2002). The performance of gesture in children with and without DCD: effects of sensory input modalities. Developmental Medicine & Child Neurology, 44: 699-705.

10. Zoia, S., Henderson, S., Barnett, A., Pelamatti, G., Marte, R., Scabar, A. (2002). A new test for the Assessment of Motor Coordination Skills: preliminary data about the reliability of the Movement Assessment Battery for Children. Psichiatria dell'Infanzia e dell'Adolescenza, 69: 129-146.

12.

Structural, laboratory and other Support for the Project
Strutture, laboratori e supporti vari per la ricerca

Trieste:
Kinematic Laboratory is part of the Child Neurology and Psychiatry Ward, Department of Pediatrics at the Institute for Maternal and Child Health, IRCCS "Burlo Garofolo" of Trieste. It hosts 8 people, comprising 3 full researchers and 1 part-time, 3 associated psychologists and 1 neurologist. The laboratory has three rooms: one completely dedicated to the kinematic equipment (Proreflex System, Qualisys Company with 4 infrared camera), another wide room is devote to the neuropsychological assessment and lastly there is one office.
The laboratory is linked to the clinical ward and the EEG and EMG laboratories, that hosts 4 Paediatrician neurologists, 1 developmental psychiatrists, 2 psychologists, 3 nurseries, 4 neurophysiopathology technicians, 3 master students and 4 medical postdoctoral fellowship in pediatric neurology and psychiatry. The Lab. has also privileged access to the Child Neurology and Psychiatry Ward's activities and spaces. The clinical activity includes all the neurological, psychiatric and neuropsychological diseases as well as all the possible developmental neurocognitive disorders which can affect children from 0 to 18 years old. The Child Neurology and Psychiatry Ward has more than 20 offices and 3 experimental Labs. The Lab has also privileged access to an fMRI scanner for developmental neurology, to the Speech Ward and the Physiotherapy Ward of the Institute for Maternal and Child Health. Researchers in the Laboratory regularly handle grants financed by the National Health Minister, the European Community and other national and international funding agencies. Part of the researchers have also collaborations with the University of Trieste.

Grottaferrata:
"Eugenio Litta" is a rehabilitation Center that operates within the Italian National Health System. It provides care and treatment in three modalities with a dedicated ward for each: Residential (total: 45 istitutionalized children), Semi-Residential (total 95 adults in Occupational Therapy), and Non-Residential (out-patient for 120 children) The numbers indicate daily average number of patients. All patients have a

developmental disorder, from Mental Retardation, to Autism, Cerebral Palsy, Epilepsy, Learning Disabilities etc. The three wards have laboratories and facilities that include Swimming pool, gym, ceramics atelier, Music Therapy room with video facility through one-way mirror, Psychomotor Therapy rooms, Physical Therapy rooms, Autism cognitive treatment rooms, Speech therapy rooms. An EEG/EP facility is also available and is run by Dr. Luisa Lopez as part of the therapeutic follow up for all patients There is a scientific-teaching agreement with the Child Neurology Department of the University of Rome Tor Vergata that provides espertise on training, and trainees in neuro-psychomotor rehabilitation, besides providing access to the University Hospital facilities for both clinical and research purposes.
The out-patient ward, where the research will be performed, hosts 4 Medical Doctors, 4 Psychologists, 12 Speech Therapists, 5 Neuro Psychomotor Rehabilitation Therapists, 1 Physical Rehabilitation Therapist, 1 Occupational Therapist, 2 Professional Educators. Through a teaching agreement with University of Rome Tor Vergata there are also 15 students, trainees in Neuro Psychomotor Rehabilitation Therapy, and through other Institutions there are 2 Psychologists, Trainees in Cognitive Behavioral Psychology.

Marseille:
The INCM is a mixed research Unit, CNRS and University of the Mediterranean. It hosts around 80-100 people, comprising full researchers, associated neurologists and psychiatrists, post-doc, PhD and master students. The laboratory has more than 50 offices and 15 experimental Labs, including 4 EEG systems, 6 primate neurophysiology recording set-ups, several eye tracking systems, 1 3D environment. The Lab has also privileged access to an fMRI scanner (dedicated to research) and to a MEG (Timone Hosp). Because there are several neurologists and psychiatrists (including pedo-psychiatrists) associated to the Lab, the Lab has also access to several types of pathologic populations (adults with schizophrenia, bipolar syndrome, aphasia, Alzheimer, Parkinson, and both children and adults with William's syndrome, autism and dyslexia). Researchers in the Laboratory regularly handle grants financed by the National French Agency, the European Community (ITN) of other national and international funding agencies (eg. HFSP).

13.

Research Budget - Please indicate distinct budgets for the first and second years of activity and detailed allocation: personnel (scolarships, consulting, etc.), reagents, equipments, publication costs, travel and meetings, other possible expenses (Note: Any substantial variations in the specific budget during the course of the research project must be approved by the Mariani Foundation's Scientific Committee and anyway only one a year). ***Please notice the Mariani Foundation does not finance overhead or institutional charges and the sum allotted to equipment may not exceed 20% of the total budget.***
Budget di ricerca analitico - Indicare la ripartizione tra primo e secondo anno di ricerca e la ripartizione dettagliata: personale (borse di studio, consulenze ecc.), spese per reagenti, strumentazione, pubblicazioni, viaggi e partecipazione a congressi, altre spese (Ogni sostanziale variazione del programma di spesa, durante lo svolgimento della ricerca, sarà sottoposta al parere del Comitato Scientifico e in ogni caso sarà possibile solo una modifica all’anno).
N.B. La Fondazione Mariani non finanzia overhead o spese istituzionali; inoltre, l'ammontare per la strumentazione non potrà essere superiore al 20% del finanziamento complessivo richiesto.

Research Budget - Please indicate distinct budgets for the first and second years of activity and detailed allocation: personnel (scolarships, consulting, etc.), reagents, equipments, publication costs, travel and meetings, other possible expenses (Note: Any substantial variations in the specific budget during the course of the research project must be approved by the Mariani Foundation's Scientific Committee and anyway only one a year).
Please notice the Mariani Foundation does not finance overhead or institutional charges and the sum allotted to equipment may not exceed 20% of the total budget.
Budget di ricerca analitico - Indicare la ripartizione tra primo e secondo anno di ricerca e la ripartizione dettagliata: personale (borse di studio, consulenze ecc.), spese per reagenti, strumentazione, pubblicazioni, viaggi e partecipazione a congressi, altre spese (Ogni sostanziale variazione del programma di spesa, durante lo svolgimento della ricerca, sarà sottoposta al parere del Comitato Scientifico e in ogni caso sarà possibile solo una modifica all’anno).
N.B. La Fondazione Mariani non finanzia overhead o spese istituzionali; inoltre, l'ammontare per la strumentazione non potrà essere superiore al 20% del finanziamento complessivo richiesto.

YEAR 1
Item, Unit Cost, Quantity, Total

PERSONNEL total cost € 43,560.00
1 Coordinator Psychologist, expert in clinical neuropsychology (consultant), 2,200.00€/month, 12 months, €26,400.00
3 Psychologists (consultant), 1,500.00 €/month, 2 months each one, € 9,000.00
2 Music Teachers (consultant), 20.00 €/hour, 102 hours each one, € 4,080.00
2 Painting Teachers (consultant), 20.00 €/hour, 102 hours each one, € 4,080.00

TRAVEL EXPENSES total cost € 6,400.00
Travel project meetings, each € 1000, 4, € 4,000.00
International conference, each € 1200, 2, € 2,400.00

WORKSHOP total cost €2,000.00
3 days workshop for teachers €2,000.00
This workshop is designed to tune music and art teachers on training

EQUIPMENT total cost € 13,418.40
Hardware total cost € 3,898.00
Sound Blaster, € 200, 4, € 800.00
Headphones, € 70, 2, € 140.00
Cd, € 0.80, 50, € 40.00
Loud speaker, € 80, 4, €320.00
Laptop, €1299, 2, € 2,598.00

Software total cost € 4,000.00
MATLAB©, € 2,000, 2, € 4,000.00

Neuropsychological assessment material total cost € 2,269.40
WISC III record form, €3.50, 113, €395.50
BVN record form, Erickson, 2005, € 0.05, 113, € 5.65
CMF record form, € 0.05, 113, € 5.65
Movement ABC Second Edition Kit, € 920.00, 2, € 1,840.00
MT record form, record form, € 0.05, 113, € 5.65
TPV Record form, € 0.05, 113, € 5.65
Word-PseudoWord Record form, € 0.10, 113, € 11.30

Training materials total cost € 3,160.00
Painting activity and home exercise items total cost € 1,000.00
Musical training items total cost € 2,160.00
THOMANN GIGBAG1 E-BASS, € 19, 2, € 38
ROLAND KC-150, € 365, 2, € 730
EPIPHONE EB-0 CH, € 159, 2, € 318
CORDIAL CT1 3 PP-SW, € 10, 2, € 20
GOLDON ALT XYLOPHON MODELL 10210, € 175, 4, € 700
MEINL HCAJ1NT CAJON, € 86, 2, € 172
MEINL MWB2BK WOOD BLOCK LARGE, € 15, 2, € 30
MILLENIUM HT200RD, € 10, 2, € 20
SONOR GTR20 TRIANGEL, € 5.5, 4, € 22
MILLENIUM HB5A HORNBEAM –HOLZ, € 4, 2, € 8
MILLENIUM BONGO SET WEIN ROT, € 39, 2, € 78
SCHLAGWERK SK50 GROOVY SHAKER (NATUR), € 12, 2, € 24

CHANCERY total cost € 2,000.00
Trieste € 1000
Grottaferrata € 1000

Total cost year 1 € 67,378.00

YEAR 2
Item, Unit Cost, Quantity, Total

PERSONNEL total cost € 55,360.00
1 Coordinator Psychologist, expert in clinical neuropsychology (consultant), 2,200.00€/month, 12 months, €26,400.00
3 Psychologists (consultant), 1,500.00 €/month, 4 months each one, € 18,000.00
2 Music Teachers (consultant), 20.00 €/hour, 137 hours each one, € 5,480.00
2 Painting Teachers (consultant), 20.00 €/hour, 137 hours each one, € 5,480.00

TRAVEL EXPENSES total cost € 6,400.00
Travel project meetings, each € 1000, 4, € 4,000.00
International conference, each € 1200, 2, € 2,400.00

INTERNATIONAL CONFERENCE total cost € 7,000.00
3 days, € 7,000.00
This 3 days conference on learning disabilities and music rehabilitation will gather all members of the project. Moreover we will invite several National and International specialist in this field.

EQUIPMENT total cost € 1,524.90

Hardware total cost € 0.00
Software total cost € 0.00

Neuropsychological assessment material total cost € 429.40
WISC III record form, €3.50, 113, €395.50
BVN record form, Erickson, 2005, € 0.05, 113, € 5.65
CMF record form, € 0.05, 113, € 5.65
MT record form, record form, € 0.05, 113, € 5.65
TPV record form, € 0.05, 113, € 5.65
Word-PseudoWord record form, € 0.10, 113, € 11.30

Training materials total cost € 1,095.50
Painting activity and home exercise items total cost € 1,000.00
Musical training items total cost € 95.50
CORDIAL CT1 3 PP-SW, € 10, 1, € 10
MEINL MWB2BK WOOD BLOCK LARGE, € 15, 1, € 15
MILLENIUM HT200RD, € 10, 1, € 10
SONOR GTR20 TRIANGEL, € 5.5, 1, € 5.50
MILLENIUM HB5A HORNBEAM –HOLZ, € 4, 1, € 4
MILLENIUM BONGO SET WEIN ROT, € 39, 1, € 39
SCHLAGWERK SK50 GROOVY SHAKER (NATUR), € 12, 1, € 12

CHANCERY total cost € 2,000.00
Trieste € 1,000.00
Grottaferrata € 1,000.00

Total cost year 2 € 72,285.00

TOTAL FINANCING REQUESTED FOR 2 YEARS: € 139,663.00

14.

National and international Collaborations foreseen for the project
Collaborazioni nazionali e internazionali previste per il progetto

Last Name, First Name, Insitute, Title and details on the above
Cognome, Nome, Istituto, Qualifica e specifiche

Schön Daniele, INMC-CNRS Marseille, France
Because D. Schön is partner of an European project on music and rehabilitation (International Training Network, FP7-PEOPLE-ITN-2008), the project will also possibly benefit of interactions and collaborations with the other partners of the network during meetings and workshops specifically organized on the beneficial effects of music on the brain.

Costantini Antonella
Pianist
National representative of the project "Nati per la musica" (Born to music), supported by the Centre for the Health of the Child, granted by Italian Association of Paediatrics and Italian Society for Musical Education; director of the laboratory of arts "G. Radole" in Trieste (Italy).
She will be a consultant for music and painting laboratories and she will furnish laboratory places for experimental training in Trieste.

Sila Alessandra
Graduated in Pedagogy
National representative of the project "Nati per leggere", supported by the Centre for the Health of the Child, granted by Italian Association of Paediatrics and Italian Association of Librarians.
She will be a consultant for planning activity of the laboratories.

15.

List of Abbreviations and Glossary of unusual terms
Lista delle abbreviazioni e glossario dei termini insoliti

ADHD, Attentional Deficit Disorders with Hyperactivity
DSM IV-TR, Diagnostic and Statistical Manual of Mental Disorders, Fourth Edition, Text Revision
IRCCS, Istituto di Ricovero e Cura a Carattere Scientifico
MBEA, Montreal Battery for Evaluation of Amusia
MD, Medical Doctor
ODD, Oppositional Defiant Disorder
RD, Reading Disorder
SLI, Specific Language Impairment

N.B.
Fellowships, recipients of fellowships or scholarships are required to send to the Mariani Foundation their C.V. and a detailed description of their research tasks within the project. Moreover, the Principal Investigator commits to send a scientific report on the project at the end of the first year after commencement.
I borsisti, una volta nominati, dovranno inviare il loro Curriculum Vitae e la descrizione analitica dei loro specifici compiti finalizzati alla realizzazione del progetto di ricerca. Inoltre, il Responsabile del progetto si impegna a inviare una relazione scientifica sull'andamento della ricerca alla fine del primo anno dall'inizio del progetto.

Please Note
The Principal Investigator commits to verify that the Mariani Foundation is acknowledged in all scientific publications that may result from the project and to see that a copy of each relevant publication is sent to the Mariani Foundation.
Il Responsabile si impegna a citare la Fondazione Mariani in tutte le pubblicazioni frutto della presente ricerca e ad inviare alla Fondazione copia delle pubblicazioni pertinenti.
