# Supplementary material for: Music Training Increases Phonological Awareness and Reading Skills in Developmental Dyslexia: A Randomized Control Trial
Source: PLoS One. 2015 Sep 25;10(9):e0138715. doi: 10.1371/journal.pone.0138715 (PMC4583182; doi:10.1371/journal.pone.0138715)
Supplement: S1 Protocol — (PDF) [file pone.0138715.s004.pdf]

ClinicalTrials.gov Protocol and Results Registration System (PRS) Receipt
Release Date: 12/09/2014

Rhythm and Music to Rehabilitate Reading Disorders (ReMus)

This study has been completed.

Sponsor:	Aix Marseille Université
Collaborators:	WHO Collaborating Centre for Maternal and Child Health, Trieste<br>University of Roma La Sapienza
Information provided by (Responsible Party):	Daniele Schon, Aix Marseille Université
ClinicalTrials.gov Identifier:	

Purpose

The purpose of this study is to determine whetehr music training is an effective treatment of reading disorders

Condition	Intervention	Phase
Reading Disability	Behavioral: Music training<br>Behavioral: Visual arts	N/A

Study Type: Interventional
Study Design: Treatment, Parallel Assignment, Open Label, Randomized, Efficacy Study
Official Title: Rhythm and Music to Rehabilitate Reading Disorders

Further study details as provided by Daniele Schon, Aix Marseille Université:

Primary Outcome Measure:

- Pseudoword reading test [Time Frame: six months] [Designated as safety issue: No]
  The primary outcome variable was the performance in the pseudoword reading test measured in terms of accuracy (percentile of number of errors).

Secondary Outcome Measures:

- others [Time Frame: six months] [Designated as safety issue: No]
  - pseudoword reading test
  - reading words and text
  - phonological knowledge
  - auditory attention
  - verbal short term memory and working memory
  - temporal processing through rhythm reproduction task, tapping task and temporal anisochrony threshold (ms)

Enrollment: 48
Study Start Date: September 2011

Primary Completion Date: June 2013
Study Completion Date: December 2013

Arms	Assigned Interventions
Experimental: Music training<br>twice a week one hour for 30 weeks, music training	Behavioral: Music training<br>This program was based on the Kodaly and Orff pedagogy and adapted to focus on rhythm and temporal processing.
Active Comparator: Visual arts<br>twice a week one hour for 30 weeks, visual arts training	Behavioral: Visual arts<br>This program emphasized visual-spatial and hand skills as well as creativity.

Detailed Description:

Introduction: Children with dyslexia show deficits in temporal processing, both in language and in music. Musical activity increases phonological awareness, word segmentation, working memory, as well as reading abilities in children with typical development, a compelling evidence for a role of music training in fostering brain plasticity. Within this theoretical framework, we investigate the hypothesis that music training, by improving temporal processing and rhythm abilities, improves phonological awareness and reading skills in children with dyslexia.

Methods: The study is a prospective, multicenter, open randomized controlled trial, consisting of test, rehabilitation and re-test Results: After rehabilitation, the music group performed better than the control group in tests assessing rhythmic abilities, phonological awareness and reading skills.

Conclusions: This is the first randomized control trial testing the effect of music training in enhancing phonological and reading abilities in children with dyslexia. The findings show that music training can modify reading and phonological abilities even when these skills are severely impaired. Through the enhancement of temporal processing and rhythmic skills, music might become an important tool in both remediation and early intervention programs.

Eligibility

Ages Eligible for Study: 8 Years to 11 Years
Genders Eligible for Study: Both
Accepts Healthy Volunteers: No

Criteria

Inclusion Criteria:

Italian native language, reading performance (accuracy and/or speed) failed on at least two of three school grade standardized Italian tests: text, words, pseudowords (cut-offs: z-score <-1.8 standard deviations from the mean for speed scores, a score <5th percentile in the accuracy scores), hearing and neurological examination within normal range, normal or corrected-to-normal visual acuity, General IQ >85 at Wechsler Intelligence Scale for Children III.

Exclusion Criteria:

Presence of comorbidity involving Attentional Deficit Disorders with Hyperactivity (ADHD), Specific Language Impairment (SLI), Oppositional Defiant Disorder (ODD), severe emotional-relational impairments, previous formal musical or painting education for more than one year, other on-going treatment. Presence of other diseases (i.e. diabetes , cystic fibrosis, asthma...) that could influence the performance in cognitive and executive functions.

More Information

Responsible Party: Daniele Schon, PhD, Aix Marseille Université
Study ID Numbers: ReMus R-11-85
Health Authority: Italy: National Bioethics Committee
